# Pan RAS-binding compounds selected from a chemical library by inhibiting interaction between RAS and a reduced affinity intracellular antibody

**DOI:** 10.1038/s41598-021-81262-z

**Published:** 2021-01-18

**Authors:** Tomoyuki Tanaka, Jemima Thomas, Rob Van Montfort, Ami Miller, Terry Rabbitts

**Affiliations:** 1 Leeds Institute of Medical Research, St James Hospital, Brenner Building, Beckett St., Leeds, LS9 7TF UK; 2 Institute of Cancer Research, Division of Cancer Therapeutics, 15 Cotswold Road, Sutton, London, SM2 5NG UK; 3 Present Address: Sanofi K.K. Tokyo Opera City Tower, Shinjuku-ku, Tokyo, 163-1488 Japan; 4 Present Address: 114 Innovation Dr, Milton Park, Abingdon, OX14 4RZ UK

**Keywords:** Drug development, Cancer, Immunology

## Abstract

Intracellular antibodies are valuable tools for target validation studies for clinical situations such as cancer. Recently we have shown that antibodies can be used for drug discovery in screening for chemical compounds surrogates by showing that compounds could be developed to the so-called undruggable RAS protein family. This method, called **A**nti**b**ody-**d**erived compound (**Abd**) technology, employed intracellular antibodies binding to RAS in a competitive surface plasmon resonance chemical library screen. Success with this method requires a high affinity interaction between the antibody and the target. We now show that reduction in the affinity (dematuration) of the anti-active RAS antibody facilitates the screening of a chemical library using an in vitro AlphaScreen method. This identified active RAS specific-binding Abd compounds that inhibit the RAS-antibody interaction. One compound is shown to be a pan-RAS binder to KRAS, HRAS and NRAS-GTP proteins with a Kd of average 37 mM, offering the possibility of a new chemical series that interacts with RAS in the switch region where the intracellular antibody binds. This simple approach shows the druggability of RAS and is generally applicable to antibody-derived chemical library screening by affording flexibility through simple antibody affinity variation. This approach can be applied to find Abd compounds as surrogates of antibody-combining sites for novel drug development in a range of human diseases.

## Introduction

Antibodies are naturally occurring, highly variable proteins that can interact with antigens with high affinity and elicit immune responses. Intracellular antibodies build on these properties but functioning within the cell allowing them to manipulate a spectrum of protein functions that is not available to antibodies per se. In this way, intracellular antibodies have been engineered in various formats ranging from whole IgG^1^, to single chain variable fragments (scFv) and to single domains (iDAbs)^2^ to bind antigens and study function. Intracellular antibodies are all together powerful tools for biological and biomedical research such as target validation but thus far, efficient methods for delivering Intracellular antibodies for drug use per se has not been achieved. While delivery is a major goal for converting Intracellular antibodies into drugs, they remain powerful tools for analysis purposes.

In the main, Intracellular antibodies are fragments of whole antibodies comprising just the variable (V) regions and do not carry the effector functions that are conferred for instance by Fc-mediated effector functions. This implies that molecules that could be identified that are surrogates of the antibody complementarity determining regions (CDRs) of the V regions. We have previously selected an intracellular antibody scFv that binds to the active-form RAS switch I region, predominantly through the heavy chain V-regions (VH, previously called Y6^3^), thereby inhibiting the protein–protein interaction (PPI) of RAS with effectors. This intracellular antibody VH domain antibody (herein called iDAb RAS) was used to validate that inhibition of PPI of RAS with effectors, such as CRAF, and was sufficient to halt cancer growth in pre-clinical models^4^. In work designed to establish that chemical surrogates of the antibody combining site could be selected using the interaction of intracellular antibody and target protein, we previously used a high affinity anti-RAS scFv in a competitive surface plasmon resonance assay (cSPR) to screen for RAS binding compounds^5^. These chemical compounds are surrogates of the antibody combining site and were designated as **A**nti**b**ody-**d**erived (**Abd**) compounds. Recently, an anti-HIV IgG was also used to guide the development HIV-binding compounds^6^.

The use of intracellular antibodies for chemical library screens, to identify compounds using cSPR, necessitated high affinity of the interaction (high k_on_ and low k_off_) between intracellular antibody and antigen. This is because we were seeking compounds that bind to the target protein where the antibody binds and whose binding would be competed by the presence of antibody^5^. In most chemical libraries, the compounds that bind to any target will have a range of interaction potencies. In order to select every binding compound (low and high affinity), we needed to be able to use an antibody with low affinity binding to its target antigen. Reduction in the antibody variable region affinity can be exploited by site-directed mutagenesis to alter affinity, without losing binding-specificity. We have employed such a process, designated dematuration, whereby residues in the V region CDRs are altered to impair affinity. We have generated a dematured version of the anti-RAS iDAb RASdm and used this antibody fragment in a chemical library AlphaScreen to obtain compounds that interact with HRAS^G12V^. One compound selected by this method is a pan-RAS interactor that binds KRAS, HRAS and NRAS proteins in their GTP-bound form with a Kd of approximately 37 mM. This in vitro adaptation of our method to use intracellular antibodies for target validation and selection of Abd compounds^5^ can be applied to any target protein that can be expressed in recombinant form and for which an antibody is available.

## Results

### Reducing affinity of antibody fragments binding to RAS proteins

**Figure 1:**
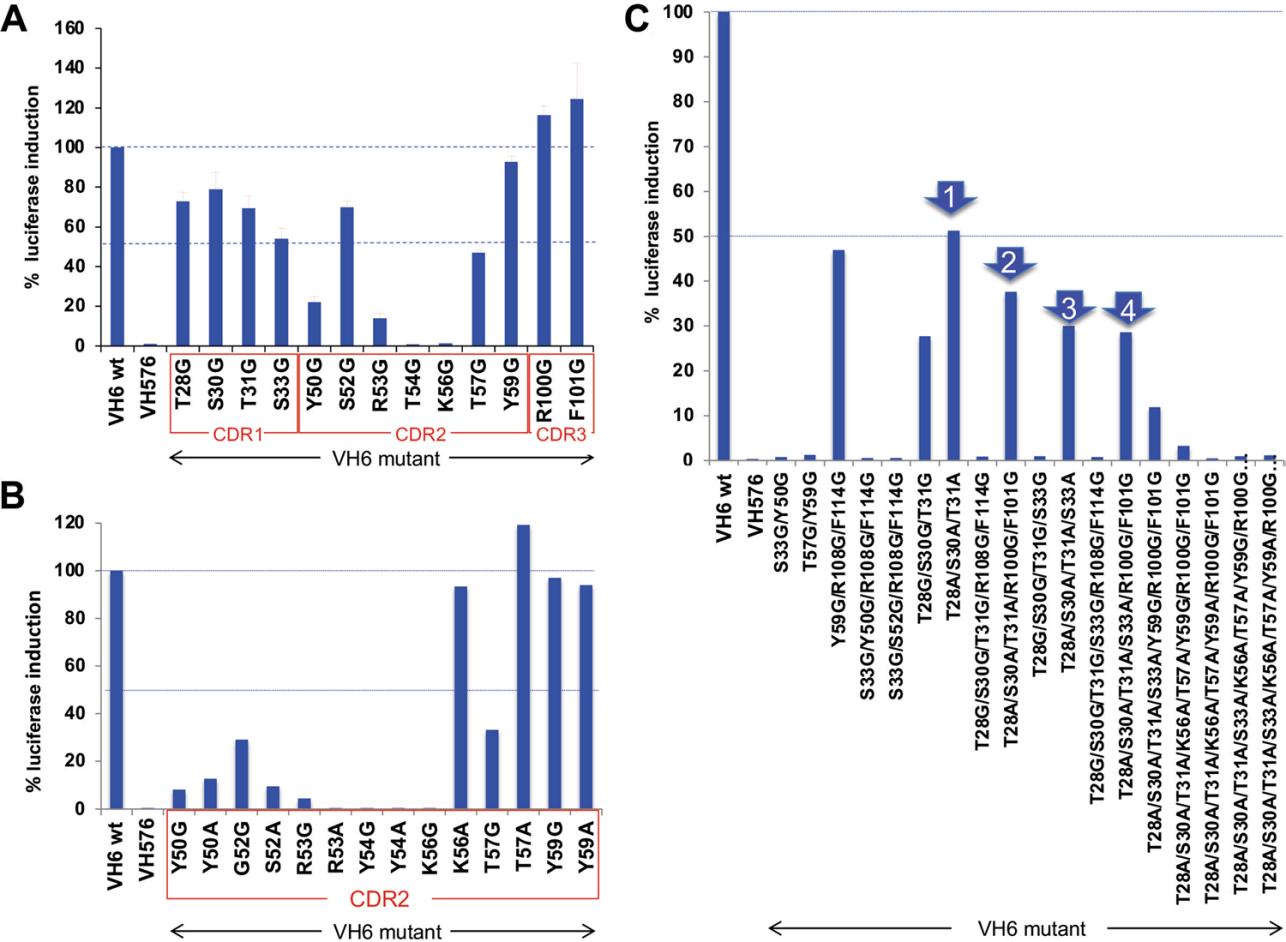
Mutagenesis of anti-RAS iDAb by glycine-alanine scanning of VH CDRs analysed with mammalian two hybrid assays. Mammalian two hybrid assays were carried out to delineate key residues for interaction between RAS and anti-RAS VH6 iDAb. The triplex plasmids^2^ with HRAS^G12V^ baits (Gal4 DBD fusion) and various anti-RAS VH6 mutant preys (VP16 fusions) were transfected into a CHO line with stable integration of the firefly *luciferase* reporter gene (CHO-luc15). Interaction signals measured by firefly luciferase gene activation are normalized to Renilla luciferase activity (assessed as percentage of luciferase activity compared to positive control using wild type unmutated anti-RAS VH6 (VH6 wt)). An anti-LMO2 VH576 iDAb was used as negative control. Panel (**A**): VH6 mutants with glycine substitution on CDR1, 2 and 3. The residue number of mutant on x-axis indicate according to IMGT numbering system (see also Supplementary Fig. 2 showing the sequence of the iDAb VH6). All the chosen mutated residues shown are known to be direct contacts to RAS proteins^3^. Panel (**B**): Glycine or alanine mutations of VH6 residues on CDR2. Panel (**C**): Effects of combining glycine and/or alanine mutations in combination from CDRs 1, 2 and 3 of iDAb RAS. The four combinations (arrowed 1–4) were used to inform the decision on the final set of mutations in the dematured iDAb RASdm.

The X-ray crystallography structure of the scFv with HRAS^G12V^-GTP showed that the VH CDRs of the scFv made the predominant interactions with RAS in the complex with a Kd of 94 pM^3^ (Supplementary Fig. 1 compare respectively the crystal structures of (A) HRAS^G12V^-VH and (B) RAS^G12V^-scFv with those of RAS bound to effectors PI3Kγ (C), CRAF (D) or RALGDS (E); all these proteins bind at the RAS switch region). The dematuration of the affinity required determination of the interaction energy of the CDR residues. This was determined for residues in the VH CDR1, 2 and 3 using a mammalian cell-based two-hybrid luciferase assay following mutation of specific residues to glycine and alanine. The assay comprised co-transfecting a DNA-binding Gal4-DBD-HRAS^G12V^ bait with various mutant anti-RAS antibodies, as iDAb-VP16 fusions, into a cell line carrying a firefly *luciferase* reporter gene^2^. Figure 1, panels A and B show the interactions of DNA binding domain (DBD)-HRAS^G12V^ with mutant iDAb-VP16 fusions and the results are normalised to 100% for DBD-HRAS^G12V^ with wild type iDAb-VP16 (the positions of the amino acids are shown in Supplementary Fig. 2). Several of the glycine or alanine changes in CDR2 had major effects on the interaction as would be expected from the crystal structure and also with some changes ablating the interaction completely (Fig. 1B).

As we aimed to retain binding specificity but with reduced affinity, we made glycine or alanine mutants that variously combined changes from combinations of the CDRs and ascertained the effects on binding in the two-hybrid luciferase assay (Fig. 1C). The mutations appear to either cause essentially complete loss of binding, such as S33G (CDR1) plus Y50G (CDR2) or about 50% reduction such as T28A plus S30A and T31A (all CDR1) (arrow 1 in Fig. 1C). By adding two CDR3 mutations to CDR1 combination R100G and F101G, the luciferase activation was reduced by a further 15–20% which was a similar reduction found by an additional CDR1 mutation, S33A (respectively indicated by arrows 2 and 3 in Fig. 1C). Combining T28A, S30A, T31A, S33A, R100G and F101G maintained the luciferase stimulation at about 30% (arrow 4 in Fig. 1C). Thus, the interaction between RAS and VH is maintained with weak affinity, even if the direct contacts between RAS switch regions and all CDR1 and 3 are diminished. This suggests that the single peptide region, which is the CDR2 loop of the anti-RAS VH is crucial for its binding ability and maintaining specificity. These data, together with the other mutation data, suggested that the combination of the four residues in CDR1 and the two mutations in CDR3 could be optimal changes in the antibody combining site to the aim of producing a useful dematured protein (iDAb RASdm). The locations of the mutations within the iDAb RAS are summarized in Supplementary Fig. 2.

**Figure 2:**
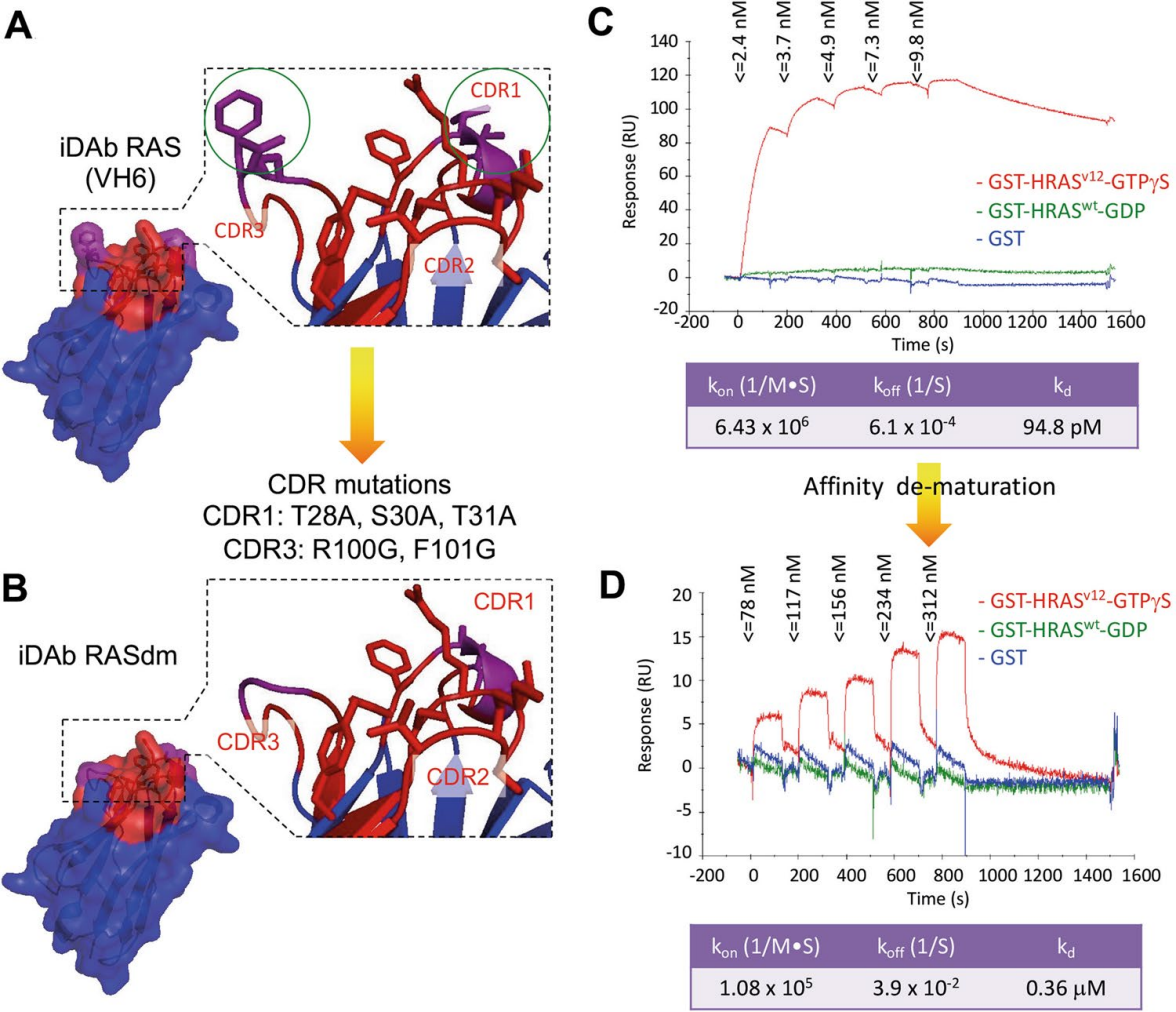
Dematuration of iDAb RAS affinity by mutagenesis of the CDR region. The most critical CDR residues of the anti-RAS iDAb VH6 were determined by the mutagenesis data shown in Fig. 1. Mutation of specific CDR1 and CDR3 residues was used to engineer the dematured iDAb. Panel (**A**, **B**) show surface representations respectively of unmutated iDAb (**A**) and the engineered iDAb (carrying combined mutations of T28A/S30A/T31A in CDR1 and R100G/F101G in CDR3) (**B**). In each, the CDRs are depicted in red and purple, substituted residues in purple and the framework region in blue. The insets are magnifications of CDRs shown in ribbon form and amino acid residues involved in the binding to RAS are shown in stick configuration. The mutated residues for the engineered iDAb are indicated in green circles in panel (**A**). Panels (**C**, **D**) show affinity measurement of anti RAS antibody (**C**) and engineered mutant (**D**) with RAS-GTPγS using surface plasmon resonance. The binding kinetics were measured using the intracellular antibody in scFv format, comprising VH6 plus VL204 held by a short linker sequence (**C**) or the mutated iDAb VH6 plus VLI21^3^ by single cycle surface plasmon resonance kinetics method on a BIAcore T100. The graphical representations are sensorgrams of the two scFvs with GST (blue sensorgrams), GST-RAS-GDP (green sensorgrams) or GST-RAS-GTPγS (red sensorgrams). The response units were normalized to the response in a channel without captured protein. The tables show values for the association rate (k_on_ M^-1^ s^-1^), dissociation rate (k_off_ s^-1^), and the equilibrium dissociation constant (K_d_) determined with the BIAcore T100 evaluation software 2.0.2.

Molecular models of the unmutated and mutated VH regions are illustrated in Fig. 2A,B respectively. The binding affinity of the iDAb RASdm was compared to the unmutated iDAb using surface plasmon resonance (SPR). An anti-RAS VH was used in an scFv format to bind immobilized RAS proteins (GST-HRAS^G12V^-GTP, GST-HRAS^G12V^-GDP, or GST-only negative control). The CDRs of the VH single domain are illustrated to show the predicted structural change that would occur due to the mutations (Fig. 2A,B). The intracellular antibodies are based on human VH Dab sequences and on a consensus framework that are well expressed in cells as iDAbs^7^. However, the Dabs do not express well in *Ecoli*-expressed recombinant form and therefore to facilitate recombinant protein expression, the VH was linked with VL in scFv format (VL121 or VL204^3^). The crystal structure of anti-RAS scFv with RAS shows that the main interactions involve the VH^3^ (Supplementary Fig. 1B illustrates the scFv-RAS interaction) and thus scFv were used for recombinant expression. As we showed previously^3^, the anti-RAS scFv only binds to RAS bound to the GTP and not to RAS-GDP and the SPR data with RAS-GTPγS shows a k_off_ of 6.1 × 10^–4^ s^-1^ characterising the sensorgrams with a shallow rate of dissociation (Fig. 2C). The K_d_ was calculated at 95 pM. The dematured protein conversely showed rapid dissociation in the SPR kinetics with k_off_ of 3.9 × 10^–2^ s^-1^ yielding a K_d_ of 0.36 μM (Fig. 2D), which is 4000 times reduction in potency. Furthermore, the direct interface of VH and RAS-GTP is minimized, specific and focused. In a chemical library screen with restricted diverse chemical matter, we would expect initial hits to have weak binding capability with the crucial binding region and especially if the objective is to obtain inhibitors of PPI. Accordingly, we considered the 0.36 μM affinity of the interaction between the target RAS and the intracellular antibody to be suited for use in a chemical screen.

### Screening in vitro using antibody fragments to derive RAS-binding Abd compounds

An AlphaScreen assay was established with GST-HRAS^G12V^-GTP bound to GST-beads and HIS-tagged scFv RASdm bound to nickel-beads. The Alpha assay comprised interaction of anti-RAS and HRAS proteins and signal from the acceptor beads generated after excitation of the donor nickel-beads (depicted in Supplementary Fig. 3A). The assay was optimized by demonstrating the ability of the high affinity unmutated scFv to compete the interaction between RAS and scFv RASdm that was first used to confirm the specific interaction of the scFv RASdm (as illustrated in the assay Supplementary Fig. 3A). 100% inhibition of scFv RASdm binding to RAS by non-mutated scFv was observed (Supplementary Fig. 3B). The interactions were optimized for protein concentrations linked to the donor and acceptor beads (Supplementary Fig. 3C,D). The interaction of HRAS^G12V^-GTP with the dematured anti-RAS scFv was robust (Supplementary Fig. 3D) yielding Z factors of 0.74–0.85 being considered suitable for high throughput screen^8^. No signal was observed when the HRAS^G12V^ was bound to GDP because the anti-RAS antibody is specific for the activated form of RAS or when the non-mutated anti-RAS was present as a competitor (Supplementary Fig. 3D), consistent with the preferential binding of the anti-RAS antibody to activated RAS and with the dominant binding of the non-mutated antibody compared to dematured form.

**Figure 3:**
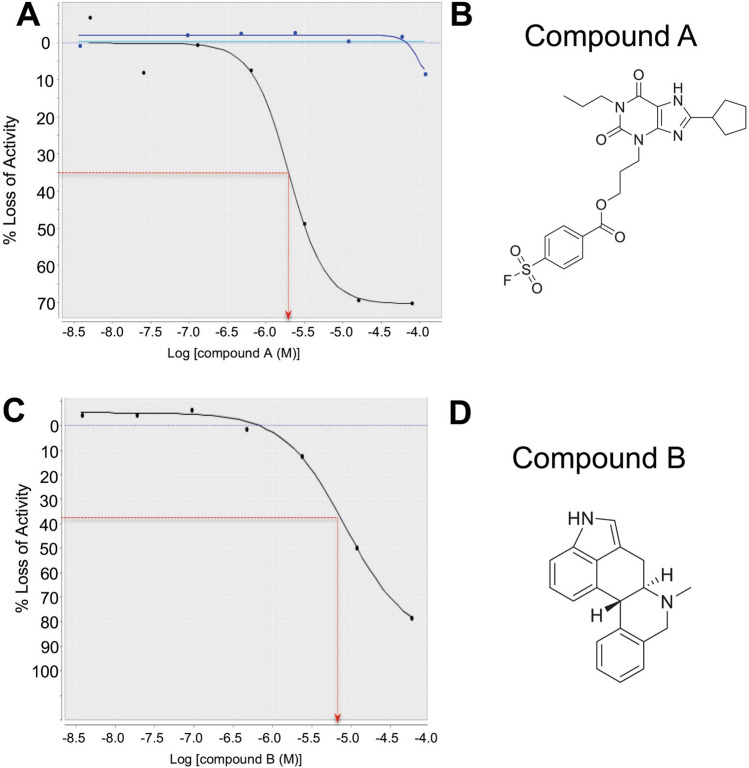
RAS hit compounds from Abd screen AlphaScreen. The Sigma-Aldrich LOPAC^1280^ of 1280 bioactive compounds collection and Tocris 1280 compounds library were screened using the AlphaScreen assay. Two compounds were selected for study using dose–response inhibition of the interaction between HRAS^G12V^-GTPγS and iDAb RASdm. Panel (**A**) shows dose response of the hit compound A (FSCPX, shown in Panel (**B**)). The blue line represents data when a non-relevant compound (a non-binder of either HRAS or antibody) is added in the assay, showing there is no interference in the AlphaAssay. Panel (**C**) shows the dose response of compound B (CY 208–243, panel D). Panel (**B**) is the chemical structure of compound A (8-Cyclopentyl-3-(3-((4-(fluorosulfonyl)benzoyl)oxy)propyl)-1-propylxanthine) and panel (**D**) is the chemical structure of compound B (indolophenanthridine or 4,6,6a,7,8,12b-hexahydro-7-methylindolo(4,3-ab)-phenanthridine). The graphs show the percentage inhibition of signal from interaction of the scFv RASdm and HRAS^G12V^-GTPγS (black lines) with increasing concentrations of compounds (compared to the activity without compounds). Compound A shows 2 µM IC_50_ and compound B shows 6 µM IC_50_ for the of binding of the dematured antibody with RAS protein.

**Figure 4:**
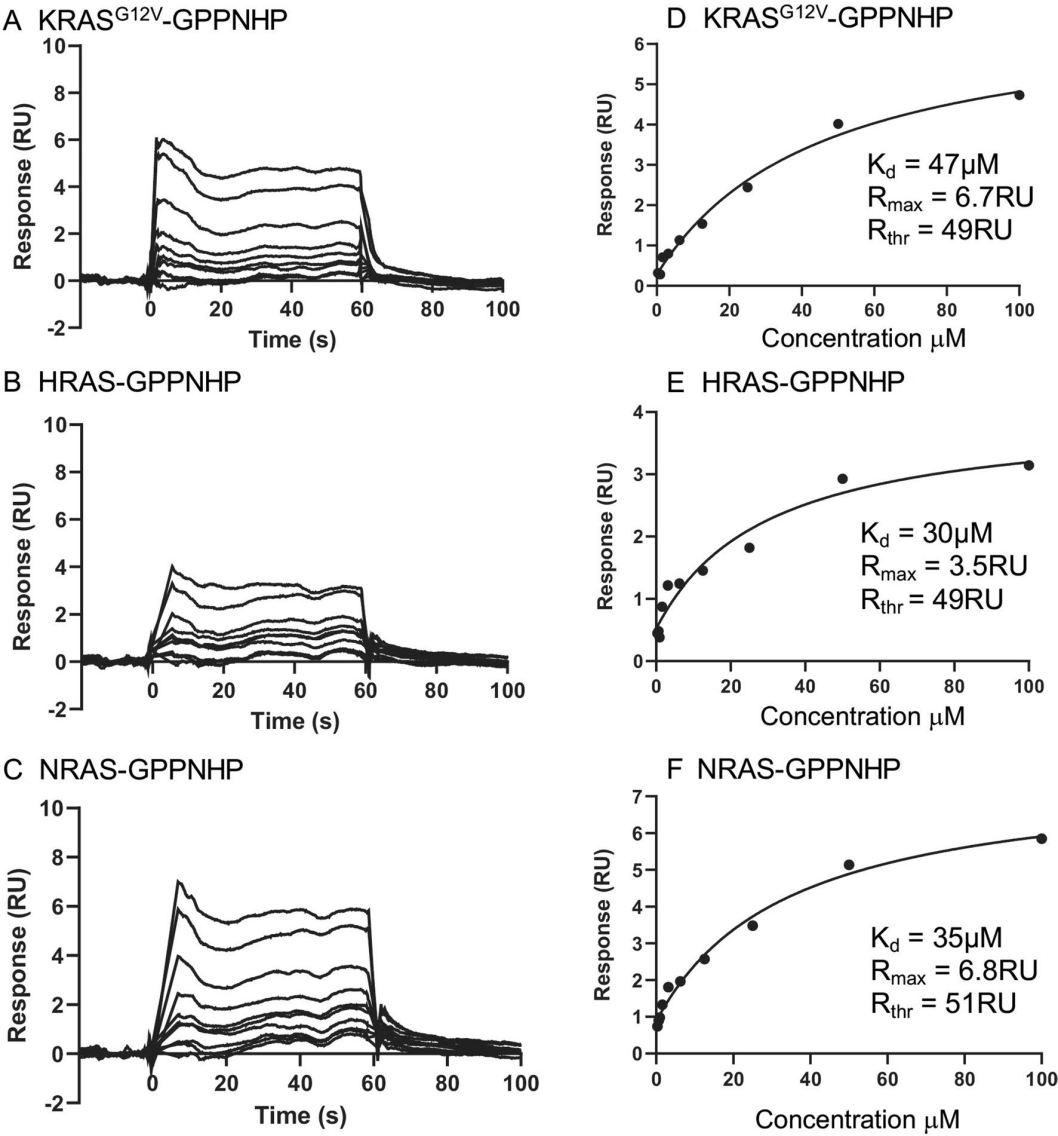
Surface plasmon resonance of compound B with the three RAS isoforms. SPR profiles were obtained using a Biacore T100 and the binding affinity of compound B was determined using biotinylated proteins bound to a streptavidin-coated chip. Flow cell Fc2 had bound biotinylated KRAS^G12V^-GPPNHP (captured RU 4163); Fc3 biotinylated HRAS-GPPNHP (captured RU 4332) and Fc4 biotinylated NRAS-GPPNHP (captured RU 4517). Flow cell 1 was used as the reference cell with no captured protein. The compound was passed over the chip at a range of concentrations from 0.312 μM to 100 μM and K_d_ values determined by plotting concentration against response and fitting to a 1:1 binding model using Biacore Evaluation software. Panels (**A**–**C**) show the sensorgrams and panels (**D**–**F**) the corresponding binding curves. Compound B binds to all three RAS-GPPNHP isoforms with similar affinity.

Two chemical libraries (Sigma-A LOPAC 1280 and TOCRIS libraries, each of 1280 compounds) were screened in this AlphaScreen assay as proof of concept, with each compound tested at a single concentration of 50 mM. Five potential hits were identified from the libraries (0.195% of the input compounds). Two commercially available compounds (designated A or Abd12 and B or Abd13) were further evaluated using a dose–response Alpha assay comparing their effect on the interaction of RAS-scFv RASdm PPI (Fig. 3) and compared to effects of non-relevant compound on the PPI (Fig. 3A). Compound A showed an IC_50_ of 2 mM and compound B of 6 mM for the inhibition of the interaction of the dematured antibody with HRAS^G12V^-GTP protein in these Alpha assays. While the mM IC_50_ depends on the amounts of donor and acceptor protein beads used in the Alpha assays, the data demonstrate dose responses of the compounds inhibiting RAS-anti-RAS interaction and are a measure of comparative efficacy of compounds A and B. The binding potency of the compounds was therefore confirmed and further analyzed using an orthogonal SPR assay, that depends on generating binding signal rather than inhibition of signal from the RAS-antibody PPI that was used to identify the hits. Accordingly, we used SPR to determine direct RAS-binding and because the anti-RAS intracellular antibody interacts with the three isoforms of activated RAS^3^, we assessed if the compounds also show a pan-RAS binding. Biotinylated KRAS, HRAS or NRAS were bound to streptavidin SPR chips and compound B was applied. We found that the compound binds to all three isoforms of RAS with similar affinity, ranging from Kd 47 mM with KRAS, 30 mM with HRAS and 35 mM with NRAS (an average K_d_ of 37 mM, Fig. 4). As expected because we used a pan-RAS antibody in the chemical screen, the compound is a pan-RAS binder. This is similar to our findings with the compounds selected using the high affinity version of the iDAb in the cSPR approach^5^.

## Discussion

### Compounds that bind RAS proteins selected using antibody-guided Abd methods

In previous work, we developed a method in which antibody binding sites were used in the screening on chemical libraries to identify compounds that are surrogates of antibody^5^. The method is called **A**nti**b**ody-**d**erived compound technology (Abd technology). In this first application^5^, a high affinity antibody fragment was used as a competitor against the binding of compounds to RAS target protein. We now describe the further development of Abd technology that relies on the inhibition of interaction antibody to the target by compounds, rather than the inhibition of binding of the compounds to the target. In order for this application to be effective, it is necessary to reduce the antibody binding affinity (or use a low affinity antibody in the first instance). By implementing an in vitro AlphaScreen interaction between the target (RAS) and a dematured (low affinity) anti-RAS antibody we were able to select new pan-RAS binding compounds.

This new version of Abd technology relies on the reduction of intracellular antibody affinity by sequential mutation of the CDRs to reduce affinity. However, it is necessary to maintain specificity. In the process of dematuration, it is difficult to assess when an antibody loses specificity. Heteroclitic antibodies that bind to an antigen that is not the original immunogen, generally involves binding to similar proteins. The loss of specificity is unlikely to be a problem with the anti-RAS antibody because it already binds to the three RAS isoforms but could be an issue for other targets that needs to be considered. In the dematuration process, the Gly/Ala scanning is crucial to find the minimum number of key CDR residues whose changes gives the required affinity reduction and in our work, three residues in CDR1 and two in CDR3 were substituted. However, while antibody-target binding must be assessed by biophysical means during dematuration, any selected chemical compounds must be analysed in depth during medicinal development to determine cross reactivity.

### Antibody-derived compounds bind the undruggable RAS proteins

We have used the RAS protein as a target for this method as this was previously regarded as undruggable^9–11^. We provide further evidence of RAS druggability and that antibody binding sites can be employed to select small molecules for drug discovery as starting chemical matter for medicinal chemistry drug development campaigns. Compound A (Fig. 3A) is a heterocycle that would require crystal soaking to facilitate structure-based drug design to place this on a formal drug development programme. This compound includes a fluorosulphonyl substituent that could covalently react with RAS or the antibody, However, we found no inhibition of interaction between the non-mutated anti-RAS scFv and HRAS^G12V^ (in Alpha assays performed with an identical protocol, unpublished data) indicating that the compound does not covalently interact with either proteins or the reagents. Further, the low Kd in SPR (Fig. 4), lack of inhibitory effect in Alpha controls (Fig. 3) argue against chemical reactivity as the reason for the Alpha assay inhibition. Compound B (Fig. 3), previously shown as a weak dopamine D1 receptor agonist^12,13^, is a complex, unfunctionalized polycyclic compound that displays a low response in SPR analysis with KRAS, HRAS and NRAS proteins, as did the initial RAS-binding Abd compound in the cSPR high affinity antibody screen^5^. This, together with the relatively poor K_d,_ indicates that compound B would require again require crystal soaking data to inform structure-based drug design to allow hit to lead strategies to be devised. The low binding strength of these initial library hits, in turn resulting from the small size of the chemical library used, would preclude meaningful cell-assays at this stage.

Structure–activity relationship studies to evolve compounds with increased potency from the initial chemical matter will await future studies. But in future application of the dematuration approach, larger chemical libraries (comprising wider diversity of chemical matter) should yield initial hits with higher binding potency. Since we initially used inhibition of target-antibody as a hit identification strategy, the approach can be made into high-throughput with large chemical libraries identifying target-binding hits, coupled to plate-based miniaturization. Further, with high-throughput screening of large libraries, it should be able to involve the original (i.e. unmutated) scFv since higher affinity compounds should by identifiable in diverse chemical sets.

The therapeutic index of pan-RAS inhibitors will depend on the effects of targeting mutant *RAS* (*KRAS, HRAS* or *NRAS*) oncogene addiction in tumour cells compared to the effect on normal cells when activated RAS is inhibited. Only sites of activated RAS (such as in the gut epithelium) are likely to be affected and it has been shown that loss of RAS from normal cells causes cessation of growth but not cell death^14^ indicating likely tolerance to pan-RAS inhibitors in normal settings. We found that protein-degradation of the KRAS isoform in KRAS-mutant tumours, comparing intracellular antibodies and DARPins, could specifically ablate these cells^15^. In future, isoform-specific antibodies would serve the purpose of potentially allowing Isoform-specific compound development and the Abd technologies will be an important potential application.

### Antibody-derived (Abd) compound methods are widely applicable to drug discovery

The work we describe further shows that the notion of non-druggability fades if new methods are applied to identify hit chemical matter. Further, we show that antibody fragment binding sites can be valuable starting points for drug discovery via selection of chemical surrogates of antibody fragment binding sites. In this case, we have used an AlphaScreen in vitro assay but other direct interaction assay such as FRET could be envisaged. We have used an intracellular antibody fragment in this case but similar approaches could be used for other macromolecules such as affimers^16^, monobodies^17^ or DARPins^18^. An advantage of antibodies or antibody fragments is that dematuration does not require structural information as the primary sequence identifies the CDRs for mutagenesis and glycine/alanine scanning can be undertaken with just the knowledge of the primary antibody sequence. Recently, a study of anti-HIV antibodies also demonstrates that chemical entities can be guided by the structure of the antibody combining site^6^.

Our primary aim was to use intracellular antibodies for target validation and subsequently screening for Abd chemical surrogates^5,19^ that could be used for developing PPI inhibitors or compounds that can be future drugs by other mechanisms such as enzyme inhibition^20^ or protein degradation^21^. Thus, the new dematuration approach can be added to methods to employ antibody binding sites for the purpose of identifying protein binding chemical matter. This approach can be adapted to different types of in vitro interaction assays applied to find compounds in novel drug development in a range of human diseases. In addition, the search for novel therapeutic compounds implementing Antibody-derived (Abd) approaches could also be applied to antibodies that bind to pathogens such as SARS-CoV-2 and to cell surface proteins if appropriate functionalities are present on the target antigens.

## Methods

### Cloning and plasmids

The Triplex vector was used for “one plasmid” mammalian two-hybrid assays (M2H)^2^. This plasmid allows expression of a prey protein-VP16 activator domain fusion protein driven from the EF1α promoter. Anti-RAS VH single domain iDAb cDNA sequences were cloned in-frame with the VP16 transactivator domain (AD) controlled by human EF1a promoter and a bicistronic mRNA comprising the Gal4 DNA binding domain (DBD)-bait fusion protein-IRES-Renilla luciferase by SV40 promoter. For glycine/alanine scanning in mammalian two hybrid assay, HRAS^G12V^ cDNA (1–166) was subcloned into the region of bait in-frame with GAL4DBD. All constructs were sequenced to confirm in-frame fusion of the inserts with fusion partners.

### Site-directed mutagenesis for glycine/alanine scanning

Mutagenesis of the complementarity determining region (CDR) residues of the anti-RAS iDAb (VH6) was performed by PCR assembly method^2^ using pEFVP16-VH6 or subsequent VH6 mutant triplex constructs as the parental template for the PCR. Each mutagenesis comprised synthesis of two overlapping PCR products using mutant oligonucleotides (step one PCR), followed by complete fragment assembly (step two PCR) and subcloning into SfiI / NotI restriction sites of the VP16 AD of the triplex vector. Step one PCR reactions contained 0.5 mM of each reverse mutagenesis oligonucleotide plus 0.5 mM the EFFP2 primer (5′- GGAGGGGTTTTATGCGATGG -3′) (a forward primer binding within the EF-1alpha promoter) or 0.5 mM of the forward mutagenesis oligonucleotide plus 0.5 mM of the VP162R primer (5′- CAACATGTCCAGATCGAA -3′) (a back primer binding within the VP16 activation sequence), 1 U KOD DNA polymerase (TOYOBO), 2 mM MgCl_2_, 0.2 mM dNTPs, 1 × KOD PCR buffer and 10 ng pEFVP16- VH6 template. PCR reactions were carried out by denaturation at 95 °C for 2 min, followed by 40 cycles of 95 °C for 20 s, 61 °C for 10 s and 70 °C for 20 s with final extension at 70 °C for 5 min. The PCR products were electrophoresed on 2% agarose gels, extracted and purified using QIAquick Gel Extractions (Qiagen). Purified PCR fragments were used as templates for step two PCR reactions. Step two PCR reactions used EFFP2 and VP162R as primers and equal amounts of the step one fragments as templates (approximately 1 ng each). Reactions were carried out under the same thermo-cycler conditions as step one PCR. The assembled PCR fragments were purified using QIAquick PCR purification kits (Qiagen), digested with SfiI and NotI, purified by agarose gel electrophoresis and cloned into the SfiI and NotI sites of the Triplex vector. The final constructs were verified by DNA sequencing.

### Mammalian two hybrid (M2H) luciferase assays

Chinese hamster ovary (CHO) cells were grown in DMEM medium with 10% foetal calf serum, penicillin and streptomycin. A Firefly luciferase reporter CHO cell line was established by co-transfecting CHO cells with linearized pG5-Fluc (a plasmid with a minimal promoter linked to five copies of the GAL4 DNA binding sequence) (Promega) and pPGK-puro^3^ plasmids using Lipofectamine 2000 (Invitrogen). Stably transfected cells were selected for 7 days using 10 mg/ml puromycin (Sigma). The CHO-Luc stable clone 15 (CHO-Luc15) was chosen for further assays^2^. For luciferase assays, the CHO-Luc15 were seeded in 12-well culture plates the day before transfection and grown until more than 90% confluent. One μg triplex vector was transfected to obtain (co-expression of a mutant form of VH6-VP16 fusion (prey) with GAL4DBD-HRAS^G12V^ (or control) bait and the Renilla luciferase for normalising transfection efficiencies) using 2 ml Lipofectamine 2000 according to the Manufacturer’s instructions. After 48 h, the cells were harvested, lysed and assayed using the Dual-Luciferase Reporter Assay System (Promega) according to the manufacturer’s instructions. The data represent a minimum of three experiments for each point and each of which was performed in duplicate. Values are normalised for stimulated Firefly luciferase levels compared with levels for transfected Renilla luciferase.

### Recombinant protein expression and purification

For preparation of recombinant GST fusion RAS proteins, pGEX-HRAS^wt^ and pGEX-HRAS^G12V^ plasmids were transformed into E. coli C41(DE3), and proteins purified using the same procedure as described^5^. Bacterial cells were cultured at 37 °C to an OD_600_ of 0.6 and induced with IPTG (isopropyl 1-thio-beta-D-galactopyranoside, final 0.1 mM) at 30 °C for 5 h. The bacteria cultures were harvested by centrifugation and the cell pellets were resuspended in 140 mM NaCl, 2.7 mM KCl, 10 mM NaH_2_PO_4_, 1.8 mM KH_2_PO_4_, 1 mM EDTA, 2 mM MgCl_2_ pH 7.4. The proteins were extracted by cell disruption (Constant Systems Ltd., UK) at 25,000 psi at 4 °C. The GST fusion proteins were purified by glutathione-sepharose column chromatography (GE Healthcare), eluting with 50 mM Tris–HCl pH8.0, 10 mM reduced glutathione, 1 mM DTT, 2 mM MgCl_2_. The eluted proteins were dialysed against 50 mM Tris–HCl pH8.0, 1 mM DTT, 2 mM MgCl_2_ and concentrated to 10 mg/ml using a Biomax-30 ULTRAFREE-15 centrifugal filter device (Millipore). To exchange endogenous guanidine nucleotide with RAS to GDP or GTP analogue, purified GST-RAS proteins were loaded with GTPγS or GDP (Sigma). The GST-RAS proteins (0.1 mM final) were diluted in Guanidine nucleotide loading buffer (20 mM Tris pH 7.4, 5 mM MgCl_2_, 10 mM EDTA, 1 mM DTT, 10% glycerol) and added GDP or GTPγS (1 mM final). After incubation at 30 °C for 30 min, 50 mM MgCl_2_ was added to stop exchange reactions. The GTP analogue loading proteins were aliquoted into 20 ul each, snap frozen and stored at – 70 °C until further experiment.

Purification of anti-RAS scFv, the plasmids pRK-HISTEV-scFv or pRK-HISTEV-VH was carried out using the same procedure as described^5^. The expression vectors were prepared by sub-cloning scFv or VH fragments into the pRK-HISTEV vector giving in-frame fusion with a 6 × histidine tag and a TEV protease site. The plasmids were transformed into C41 (DE3), cultured at 37 °C to an OD_600_ of 0.6 and induced with IPTG (final 0.5 mM) at 16 °C for 12 h. The scFv protein was extracted from bacteria pellets with the extraction buffer (25 mM Na phosphate, pH7.4, 500 mM NaCl, 20 mM imidazole) using a cell disrupter at 25,000 psi at 4 °C and purified using His-Trap Ni-affinity columns (GE Healthcare) employing gradient elution (20–300 mM imidazole). Further purification of the protein to remove HIS-tag peptide was performed by treating with TEV protease and dialysing in 20 mM Tris–HCl pH8.0, 300 mM NaCl, 20 mM imidazole at 4 °C overnight. The scFv was purified finally by passing through a Ni–NTA agarose column (Qiagen) and by gel filtration on a HiLoad Superdex-75 column (GE Healthcare) in 20 mM Tris–HCl pH8.0, 250 mM NaCl and concentrated to 10 mg/ml for storage.

### Surface plasmon resonance (SPR) assay for intracellular antibody dematuration

The BIAcore T100 (GE Healthcare) was used to measure the binding kinetics of single chain Fv or single domain VH with RAS protein. A polyclonal goat anti-GST antibody (GE Healthcare) was immobilised on a CM5 sensorchip (GE Healthcare) by amine coupling. To immobilize the antibody on CM5 chip, the chip was first activated by flowing 100 µl mixture of 0.2 M EDC (N-ethyl-N-(dimethylaminopropyl) carbodiimide hydrochloride) and 0.05 M NHS (N-hydroxysuccinimide) at 10 µl/min flow rate. 100 µg/mL anti-GST antibody in 10 mM sodium acetate, pH 5.0 was injected at 5 ul/min for 900 s and immobilized until 15,000–25,000 RU. After immobilization the chip was immediately inactivated by injecting 1 M ethanolamine, pH 8.5 at 10 µl/min for 600 s. 5 µg/mL recombinant GST, GST-HRAS-GTPγS or GST-HRAS-GDP proteins were injected for trapping on the chip through the immobilised anti-GST antibody. The binding experiments were performed by injection of purified scFv or VH (1–400 nM) in HBS-P buffer (Biacore) containing 10 mM HEPES, pH 8.0, 150 mM NaCl and 0.005% Surfactant P20 with 1 mM MgCl_2_ at 25 °C. The RAS and scFv or VH captured chip surface were regenerated by rinsing with 40 µl 10 mM glycine–HCl at 20 µl/min flow rate. The kinetics rate constants, k_on_ and k_off_ were evaluated using the BIA evaluation 2.1 software by manufacture and the Kd values were calculate from k_off_ and k_on_ rate constants (K_d_ = k_off_/k_on_).

### Surface plasmon resonance (SPR) assay using RAS isoforms

KRAS^G12V^, HRAS^wt^ and NRAS^wt^ (all amino acids 1–166) were expressed in E.coli C41 after cloning in the pRK-HIS-TEV-Avi vector, and purified as described using^19^. The protein extracts were loaded with GPPNHP (a non-hydrolysable GTP analogue) as described^19^ and biotinylated by incubation with BirA overnight at 4 °C. Final purification was carried out by gel filtration through Superdex 75. SPR experiments were performed using BIAcore T100 (GE Healthcare). The biotin-KRAS[G12V]-GPPNHP, Biotin-HRAS-GPPNHP and Biotin-NRAS-GPPNHP were immobilised on a streptavidin-coated SA chip (GE Healthcare). The chip was prepared with 3 × 30 s injections of 1 M NaCl/50 mM NaOH at 10 µL/min flow rate, before biotinylated RAS proteins were injected at 25 µg/mL with 10 µL/min flow rate until 4000–5000 RU protein was captured. The immobilisation buffer consisted of 10 mM HEPES pH 8.0, 150 mM NaCl, 1 mM MgCl_2_. Compound binding experiments were performed at 25 °C using multi-cycle injections of compound at 10 concentrations between 0.312–100 µM. The flow rate was 30 µL/min in running buffer consisting of 10 mM HEPES pH 8.0, 150 mM NaCl, 1 mM MgCl_2_, 0.05% Surfactant P20, 5% DMSO. Data were reference subtracted and DMSO solvent correction applied, before being fitted to a Steady State Affinity 1:1 binding model using Biacore T200 Evaluation Software version 2.0.

### AlphaScreen assay

The AlphaScreen assay (Perkin Elmer) was performed in a 8 μl final volume in 1536-well white microtiter plates (Greiner). The reaction buffer contained 50 mM HEPES (pH 7.5), 100 mM NaCl, 5 mM MgCl2, 0.005% (v/v) Tween-20, 0.1% (w/v) BSA, 1% dimethylsulphoxide (DMSO) and 10 mg/ml GSH-coupled AlphaLISA acceptor beads (PerkinElmer)). Three μl/well of purified GST-RAS-GTPγS (25 nM final) and GSH-acceptor beads (10 μg/ml final, Perkin Elmer) in the reaction buffer, 1 μl/ well of each compound (various final concentrations) diluted in reaction buffer. 1 μl/ well of His-tagged dematured anti-RAS scFv#6 comprising VH6 with T28A/S30A/T31A on CDR1 and R100G/F101G on CDR3, VLI21 and flexible linker peptide (250 nM final) were added and incubated for 60 min at room temperature (RT) . Afterwards, 3 μl per well of nickel chelate–coated donor beads (10 mg/ml) were added and incubation was continued for an additional 30 min at RT. Exposure of the reaction to direct light was avoided and the emission of light from the acceptor beads was measured in the EnVision plate reader (Perkin Elmer) and analyzed using the EnVision manager software.

## Supplementary Information

Supplementary Information.
