# Supplementary material for: Pan RAS-binding compounds selected from a chemical library by inhibiting interaction between RAS and a reduced affinity intracellular antibody
Source: Sci Rep. 2021 Jan 18;11:1712. doi: 10.1038/s41598-021-81262-z (PMC7814043; doi:10.1038/s41598-021-81262-z)
Supplement: Supplementary file 1 — Supplementary Information. [file 41598_2021_81262_MOESM1_ESM.pdf]

Pan RAS-binding compounds selected from a chemical library by inhibiting interaction between RAS and a reduced affinity intracellular antibody

Tomoyuki Tanaka, Jemima Thomas, Rob Van Montfort, Ami Miller & Terry Rabbitts

Supplementary figures

Supplementary Figure 1: RAS-anti-RAS structure compared to RAS- effector RBD structure

Supplementary Figure 2: The sequence of the anti-RAS iDAb

Supplementary Figure 3: 1536-well AlphaScreen optimization

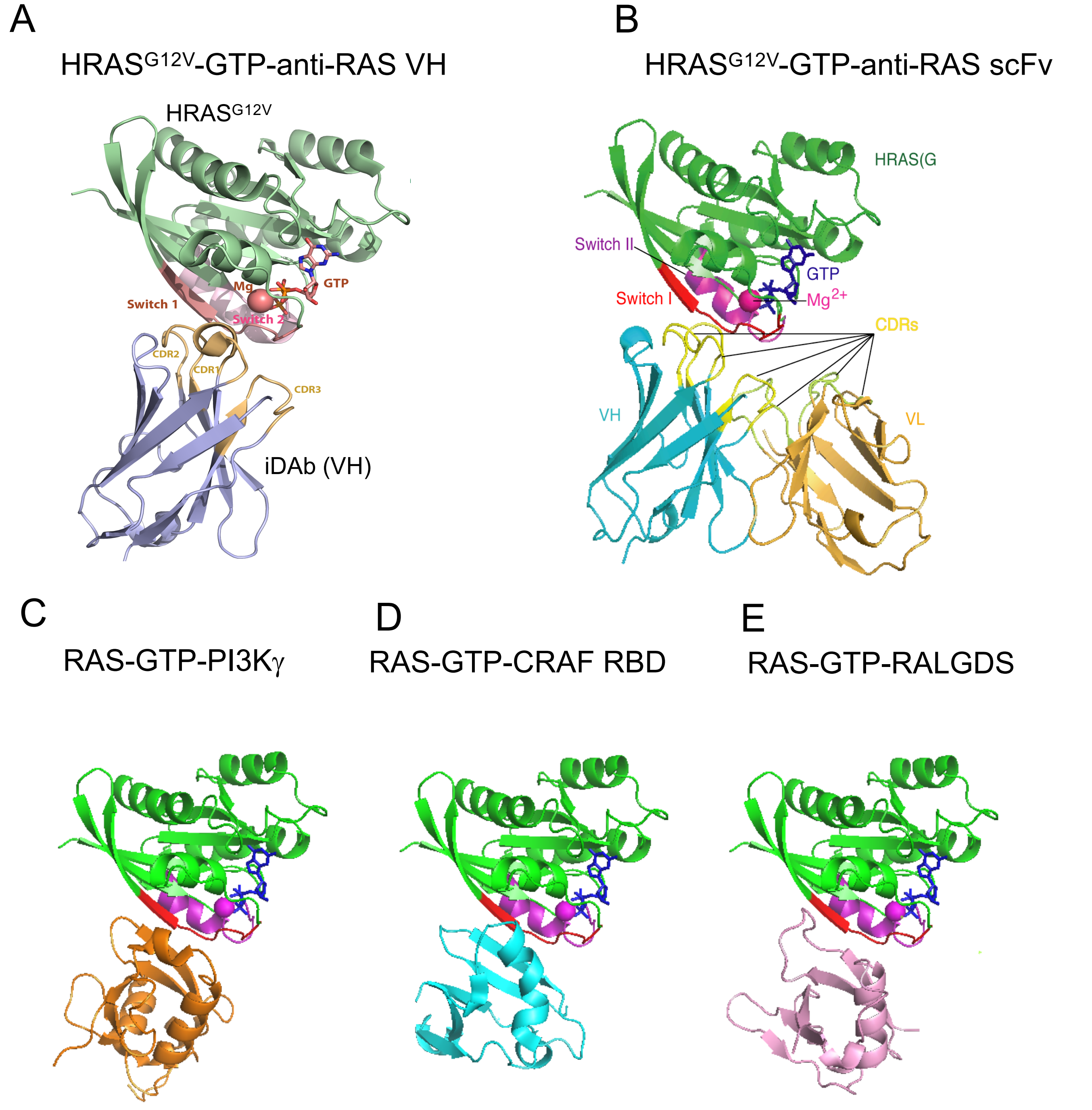

Supplementary Figure 1: RAS-anti-RAS heterodimer structure compared to RAS- effector RBD structure

Panel A: Ribbon representation of HRAS^{G12V}-GTP-anti-RAS iDAb VH6 interaction. HRAS is green, VH is purple.

Panel B: Ribbon representation of HRAS^{G12V}-GTP-anti-RAS iDAb VH6 interaction. HRAS is green, VH is purple.

Panel C: Ribbon representation of RAS-GTP bound to PI3Kγ. HRAS is green, PI3Kγ in brown.

Panel D: Ribbon representation of RAS-GTP bound to CRAF RBD. HRAS is green, CRAF is cyan.

Panel E: Ribbon representation of RAS-GTP bound to RALGDS RBD. HRAS is green, RALGDS is purple.

Adapted from ***EMBO J.* 26,** 3250-3259, 2007 DOI: 10.1038/sj.emboj.7601744

```
        10        20        30        40        50        60        70        80        90       100
ATGGCCGAGGTGCAGCTGTTGGAGTCTGGGGGAGGCTTGGTACAGCCTGGGGGGTCCCTGAGACTCTCCTGTGCAGCCTCTGGATTCACCTTTAGTACCT
TACCGGCTCCACGTCGACAACCTCAGACCCCCTCCGAACCATGTCGGACCCCCCAGGGACTCTGAGAGGACACGTCGGAGACCTAAGTGGAAATCATGGA
 M  A  E  V  Q  L  L  E  S  G  G  G  L  V  Q  P  G  G  S  L  R  L  S  C  A  A  S  G  F  T  F  S  T>    CDR1
                                                                                        28    30 31
                                                                                        A     A. A

       110       120       130       140       150       160       170       180       190       200
TTAGCATGAACTGGGTCCGCCAGGCTCCAGGGAAGGGGCTGGAGTGGGTTTCATACATTAGTAGGACGTCGAAGACGATATACTATGCAGACTCTGTGAA
AATCGTACTTGACCCAGGCGGTCCGAGGTCCCTTCCCCGACCTCACCCAAAGTATGTAATCATCCTGCAGCTTCTGCTATATGATACGTCTGAGACACTT
F  S  M  N  W  V  R  Q  A  P  G  K  G  L  E  W  V  S  Y  I  S  R  T  S  K  T  I  Y  Y  A  D  S  V  K>  CDR2
   33                                                 50    52 53 54    56 57    59

       210       220       230       240       250       260       270       280       290       300
GGGCCGATTCACCATCTCCAGAGACAATTCCAAGAACACACTGTATCTGCAAATGAACAGCCTGAGAGCCGAGGACACGGCTGTCTATTACTGTGCGAGA
CCCGGCTAAGTGGTAGAGGTCTCTGTTAAGGTTCTTGTGTGACATAGACGTTTACTTGTCGGACTCTCGGCTCCTGTGCCGACAGATAATGACACGCTCT
  G  R  F  T  I  S  R  D  N  S  K  N  T  L  Y  L  Q  M  N  S  L  R  A  E  D  T  A  V  Y  Y  C  A  R>. CDR3

       310       320       330       340       350
GGGAGATTCTTTGACTACTGGGGCCAGGGAACCCTGGTCACCGTCTCGAGC
CCCTCTAAGAAACTGATGACCCCGGTCCCTTGGGACCAGTGGCAGAGCTCG
 G  R  F  F  D  Y  W  G  Q  G  T  L  V  T  V  S  S>
   100 101
    G  G
```

Supplementary Figure 2: The sequence of the anti-RAS iDAb

The nucleotide sequence of the anti-RAS iDab VH6 and the derived protein translation is shown. The CDRs are highlighted in yellow, the numbers of the residues used in the mutagenesis shown in Figure 1 are in red and the location of the amino-acid changes in the final dematured antibody fragment are shown in blue.

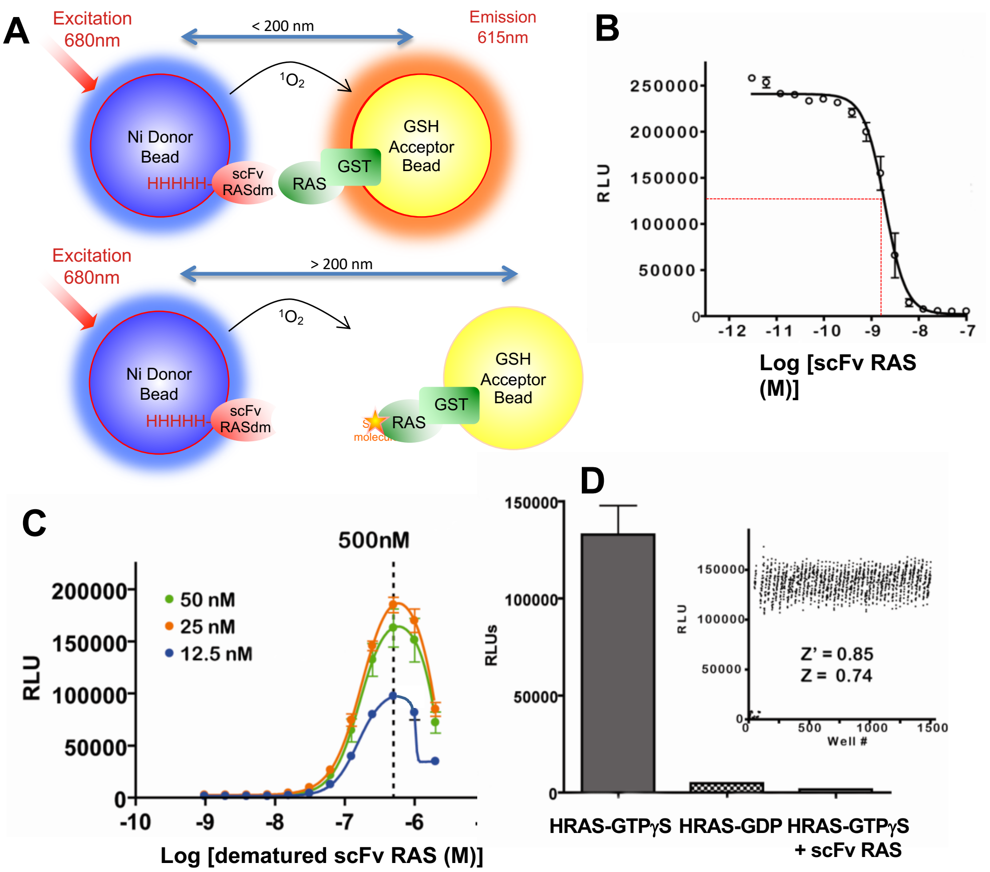

Supplementary Figure 3: 1536-well AlphaScreen optimization

Panel A: AlphaLISA glutathione (GSH) acceptor beads were used to capture GST-HRAS loaded with GTPγS (Ag). Compounds are added followed by the addition of the His tagged dematured scFv RAS, comprising engineered VH#6 RAS) plus VLI21 fragment that binds to activated RAS. Following incubation, nickel (Ni) chelate donor AlphaLISA beads were added and the chemiluminescence signal detected on an PE Envision plate reader. When interact with HRAS- GTPγS and scFv RASdm, donor and acceptor beads occurs within 200 nm in solution. The donor beads contain a photosensitizer which covers ambient oxygen to a reactive form of singlet oxygen ($^1O_2$) with a limited lifetime upon illumination at 680 nm (excitation). $^1O_2$ can only diffuse approximately 200 nm distance and energy is transferred to acceptor beads, subsequently culminating in light production at 615 nm (emission). Panel B shows the interference of binding of scFv RASdm with RAS by the unmutated scFv RAS by destroying the interaction, subsequently and separating the beads by more 200 nm, reducing emission at 680 nm excitation. Panel **C**. Optimization of interacting partners shows a characteristic "hook-effect" above 500 nM of the scFv RASdm, due to excess of the scFv and a drop-in signal with 12.5 nM, 25 nM or 50 nM RAS-GTPγS. The Alpha signal increases with increasing concentration of the scFv RASdm at constant concentration of RAS-GTPγS, but at 500 nM, the signal begins to decrease with increasing concentration of the scFv RASdm. At the point the donor beads could be saturated with His-tagged scFv RASdm and excess could disrupt association between donor and acceptor beads. For the further assay, 250 nM engineered scFv#6 and 25 nM RAS-GTPγS using 10 µg/mL acceptor and donor beads were used. Potent (2 nM) inhibition is observed for the high affinity competitor scFv RAS, comprising wild type iDAb plus VL204 supporting assay sensitivity of the assay. **Panel D**. Histogram of luminescence in control wells from a 1536-well plate using scFv RASdm and either with RAS^{wt} loaded with GTP (1), with RAS^{wt} loaded with GDP (inactivated form) (2) or scFv RASdm plus RAS^{wt} loaded and containing scFv RAS competitor. Inset shows a scattergram of the signal obtained in the 1536-well format; Z-factors, 0.74 and Z'-factors, 0.85 supporting this as a robust assay with a high signal and low signal to noise.
